# Bta-miR-10b Secreted by Bovine Embryos Negatively Impacts Preimplantation Embryo Quality

**DOI:** 10.3389/fgene.2019.00757

**Published:** 2019-08-22

**Authors:** Xiaoyuan Lin, Krishna Chaitanya Pavani, Katrien Smits, Dieter Deforce, Björn Heindryckx, Ann Van Soom, Luc Peelman

**Affiliations:** ^1^Department of Nutrition, Genetics and Ethology, Faculty of Veterinary Medicine, Ghent University, Ghent, Belgium; ^2^Reproduction, Obstetrics and Herd Health, Ghent University, Ghent, Belgium; ^3^Laboratory for Pharmaceutical Biotechnology, Faculty of Pharmaceutical Sciences, Ghent University, Ghent, Belgium; ^4^Department for Reproductive Medicine, Ghent University Hospital, Ghent, Belgium

**Keywords:** bovine embryos, secreted miRNAs, miR-10b, *HOXA1*, DNA methylation, apoptosis

## Abstract

In a previous study, we found miR-10b to be more abundant in a conditioned culture medium of degenerate embryos compared to that of blastocysts. Here, we show that miR-10b mimics added to the culture medium can be taken up by embryos. This uptake results in an increase in embryonic cell apoptosis and aberrant expression of DNA methyltransferases (*DNMTs*). Using several algorithms, Homeobox A1 (*HOXA1*) was identified as one of the potential miR-10b target genes and dual-luciferase assay confirmed *HOXA1* as a direct target of miR-10b. Microinjection of si-*HOXA1* into embryos also resulted in an increase in embryonic cell apoptosis and downregulation of *DNMTs*. Cell progression analysis using Madin–Darby bovine kidney cells (MDBKs) showed that miR-10b overexpression and *HOXA1* knockdown results in suppressed cell cycle progression and decreased cell viability. Overall, this work demonstrates that miR-10b negatively influences embryo quality and might do this through targeting *HOXA1* and/or influencing DNA methylation.

## Introduction

MiRNAs, small non-coding RNAs, function as crucial (epigenetic) regulators that can be transferred between cells (Valadi et al., 2007). MiRNAs’ selective secretion and high stability (resistant to RNase digestion and other harsh conditions) (Luo et al., 2009; Donker et al., 2012) make them good candidates as non-invasive biomarkers for preimplantation embryo quality assessment and thus increase efficiency and reduce both the risks and the costs associated with assisted reproductive treatment (ART) (Homer et al., 2017).

In a previous study, we identified 114 known and 180 novel secreted miRNAs present in bovine embryo culture media (CM). Of these miRNAs, miR-30c and miR-10b were much more abundant in CM of slow-cleaving embryos compared to intermediate-cleaving embryos. We further demonstrated that miR-30c directly targets Cyclin-dependent kinase 12 mRNA and downregulates several DNA damage response (DDR) genes (Lin et al., 2019). MiR-10b was also shown to be more abundant in the culture medium of degenerate embryos compared with that of blastocysts, and more abundant in the culture medium of slow-cleaving embryos compared with that of intermediate-cleaving embryos, indicating that overexpression of miR-10b has a negative influence on preimplantation embryo development in cattle (Lin et al., 2019). Previously, miR-10b has been shown to regulate cell invasion, apoptosis, viability, and migration in multiple cell lines in human, mouse, and goat (Chen et al., 2016; Li et al., 2016; Peng et al., 2016; Zhen et al., 2016; Zhu et al., 2016; Tan et al., 2018).

Among the possible miR-10b target genes we identified using several computational methods, *HOXA1* stood out as it was previously shown to be involved in cell proliferation in human epithelial cells (Bitu et al., 2012) and cell growth, invasion, and migration in esophageal cancer cells (Li et al., 2017). MiR-10b is located within an intron of *HOXD4* (Homeobox D4) in bovine (NC_037329.1) and mouse (NC_000068.7), and between *HOXD4* and *HOXD8* (Homeobox D8) in human (NC_000002.12). Human miR-10b has also been shown to target *HOX* genes (homeobox transcription factor) such as *HOXD10* (Liu et al., 2012; Nakayama et al., 2013) and *HOXB3* (Chen et al., 2016), thus regulating cell invasion, migration, proliferation, and apoptosis. *HOXA1* is a conserved member of the HOX family, which regulates cell fate, early development patterns, and organogenesis (Shah and Sukumar, 2010; Rezsohazy et al., 2015). As the first HOX gene to be expressed in connection with gastrulation during embryogenesis, *HOXA1* plays important roles in modulation of cell proliferation, metastasis, and invasion (Bitu et al., 2012; Zha et al., 2012; Wardwell-Ozgo et al., 2014; Taminiau et al., 2016). These diverse functional roles of *HOXA1* appear to be at least partially related to its ability to influence key signaling pathways involved in regulating the cell cycle.

In addition to miRNAs, DNA methylation, a major component of the epigenome, is also a regulator of mammalian embryogenesis (Santos et al., 2002; Zhang et al., 2016). It was previously reported that there might be a possible synergy between miRNA and DNA methylation of cancer-related genes (Shivakumar et al., 2017). To be more specific, miRNAs regulate DNA methylation by modulating *DNMTs* or methylation-related proteins (Wang et al., 2017). DNA methylation involves the covalent addition of a methyl group to the 5-carbon position of cytosine by *DNMTs* and regulates gene transcription without changing the DNA sequence (Wu and Zhang, 2014). There are three major *DNMTs*: *DNMT3a*, *DNMT3b*, and *DNMT1*. *DNMT3a* and *DNMT3b* are *de novo* methyltransferases that establish the initial DNA methylation patterns, while *DNMT1* is the maintenance DNA methyltransferase that is the most abundant DNMT in various cell types (Jeltsch, 2002; Jin and Robertson, 2013). DNA methylation plays important roles in mammalian development, X chromosome inactivation, genomic integrity, and genomic imprinting. Aberrant DNA methylation has been implicated in a lot of disease conditions, such as neurological disease, cancer, and cardiovascular diseases (Robertson, 2005; Kanai and Hirohashi, 2007; Stenvinkel et al., 2007; Bergman and Cedar, 2013). DNA methylation status has also been linked to cell apoptosis and cell proliferation (Wang et al., 2016; Loginov et al., 2017). However, possible mechanisms of synergistic interactions between miRNA and DNA methylation on transcriptomic changes in bovine embryos and its association with pregnancy outcome are so far unknown.

For this study, we hypothesized that miR-10b would exert its detrimental effect on embryo development through influencing DNA methylation and/or directly targeting certain genes such as *HOXA1*. To test this hypothesis, we supplemented culture medium with miR-10b mimics and microinjected si*HOXA1* into embryos during *in vitro* bovine preimplantation embryo development and measured *DNMTs* mRNA levels.

## Methods and Materials

### Experiment Design

In this study, miR-10b mimics were supplemented into presumed zygotes *in vitro* and embryos were cultured until day 8. Blastocysts were evaluated by morphological assessment and apoptosis staining. RT-qPCR was performed to determine the uptake of mimics by embryos and the expression of *DNMTs*. *HOXA1* was validated to be a direct target of miR-10b with dual-luciferase assay in embryos and MDBKs. To functionally study HOXA1, siHOXA1 was microinjected into presumed zygotes and embryos were cultured until day 8. Blastocysts were evaluated using similar parameters to miR-10b functional analysis. In addition, MDBKs were transfected with miR-10b mimics or siHOXA1. Cell viability and cell cycle were analyzed using WST-1 assay and PI staining.

### MiR-10b Mimics Supplementation

All animal handlings were approved by the Ethical Committee of the Faculty of Veterinary Medicine (EC2013/118) of Ghent University. All methods were performed in accordance with the relevant guidelines and regulations. The rationale behind this experiment was to investigate whether miR-10b present in the culture medium can effectively be taken up by embryos and thus affect early embryo development. To this end, bovine embryos were produced according to the previously used routine *in vitro* fertilization (IVF) methods in our lab (Wydooghe et al., 2014). Briefly, to obtain cumulus oocyte complexes from 4- to 8-mm-diameter follicles, ovaries were collected from a slaughterhouse and aspirated with a needle and fluid was pooled. Cumulus oocyte complexes were then cultured in groups of 60 in 500-μl maturation media-containing TCM199 (Life Technologies, Ghent, Belgium) supplemented with 20% heat-inactivated fetal bovine serum (FBS) (Biochrom AG, Berlin, Germany) at 38.5°C in 5% CO_2_ in the air. After 22 h, frozen–thawed bovine spermatozoa were separated using a Percoll gradient (GE Healthcare Biosciences, Uppsala, Sweden). The final sperm concentration for fertilization was 1 × 10^6^ spermatozoa/ml. After 21 h, presumed zygotes were transferred to 50-µl drops of synthetic oviductal fluid (SOF) supplemented with ITS (5 µg/ml insulin + 5 µg/ml transferrin + 5 ng/ml selenium) and 4 mg/ml BSA. MiRNA mimics (double-stranded, chemically synthesized RNAs that mimic mature endogenous miR-10b) or control mimics (double-stranded, chemically synthesized RNAs that have no homology to any known microRNA or mRNA sequences) were purchased from Qiagen (Germantown, USA) and supplemented into the culture medium of presumed zygotes with a final concentration of 1 µM. Culture occurred in groups of 25, covered with mineral oil at 38.5°C in 5% CO_2_, 5% O_2_, and 90% N_2_. Embryo quality was assessed during development and all blastocysts (day 8) were collected for RNA and immunofluorescence analysis.

### TUNEL Staining

TUNEL staining was performed using a previously described protocol (Ortiz-Escribano et al., 2017) with a commercial *in situ* cell death detection kit (Sigma, St. Louis, USA). Blastocysts were fixed in neutral buffered 4% paraformaldehyde at room temperature (RT) for 1 h, and then permeabilized with 0.1% Triton X-100 at RT for 10 min. Afterwards, blastocysts were incubated with 20 µl of TUNEL mixture for 1 h at 37°C and subsequently washed three times in phosphate-buffered saline (PBS) and finally stained with 10 µg/ml 4′,6-diamidino-2-phenylindool (DAPI) for 10 min. Slides were examined using a 20× water immersion objective on a Leica TCS-SP8 X confocal microscope (Leica Microsystems, Wetzlar, Germany). The apoptosis ratio was expressed as the total number of TUNEL-positive cells relative to the total number of the cells per blastocyst.

### Microinjection

The microinjection was performed using the previously described protocol (Goossens et al., 2010; Vandenberghe et al., 2018). Briefly, bovine zygotes were produced *in vitro* and randomly divided into three groups: (Valadi et al., 2007) a control group of zygotes that was not manipulated (Donker et al., 2012), a test group of zygotes that were injected with the short-interfering RNA (siRNA) targeting *HOXA1* (Luo et al., 2009), and zygotes injected with a non-target control siRNA (siNTC) (Qiagen, Germantown, USA). The injections were performed on an inverted microscope (Olympus, Tokyo, Japan) using piezo drill assisted micromanipulation (Narishige, London, UK). During injection, zygotes were kept at 38°C in 5-μl droplets of Hepes-buffered TCM-199 covered with mineral oil. Ten picoliters of siRNA (20 µM) was injected into the cytoplasm of zygotes 21 h post insemination (hpi). Subsequently, zygotes were washed with SOF and then cultured in groups of 25 in 50-μl droplets of SOF covered with mineral oil at 38.5°C in 5% CO_2_, 5% O_2_, and 90% N_2_. Embryo survival was checked after injection, and the cleavage rate (48 hpi) and the percentage of blastocysts [8 days post insemination (dpi)] were determined. Blastocysts were collected for RNA and for immunofluorescence analysis. Three replicates (*n* = 25 each) were performed.

### RNA Isolation and RT-qPCR.

The expression patterns of *HOXA1* and *DNMTs* were analyzed using RT-qPCR. Total RNA was isolated from three pools of five blastocysts each using the RNeasy Micro kit (Qiagen, Germantown, USA) and reverse transcribed using the iScript cDNA synthesis kit (BioRad, Brussels, Belgium). RT-qPCR was performed on a BioRad CFX 96 PCR detection system by mixing 2.5 μl of template cDNA with 5 μl of Sso Advanced SYBR Green Supermix (BioRad, Brussels, Belgium) and 300 nM of each primer in a 10-μl total volume. The PCR program consisted of an initial denaturation step at 95°C for 3 min, followed by 40 cycles of denaturation at 95°C for 5 s and a combined primer annealing-extension step at specific primer annealing temperatures for 30 s. A melting curve was produced afterwards by heating samples from 70°C to 95°C in 0.5°C increments for 5 s to confirm a single specific peak for each pair of primers (**Supplementary Table S1**). *GAPDH* and *YWHAZ*, previously shown to be stable in bovine embryos (Goossens et al., 2005), were quantified to normalize mRNA expression levels using geNorm (Vandesompele et al., 2002). RT-qPCR reactions were performed in triplicate, and the 2^−∆∆Ct^ method was used to analyze the data. The primer sequences used for RT-qPCR are listed in **Supplementary Table S1**.

The expression pattern of miR-10b was analyzed using RT-qPCR. MiRNA was isolated from three pools of five blastocysts each using the miRNeasy Mini kit (Qiagen, Germantown, USA) and reverse transcribed using a miScript II RT kit (Qiagen, Germantown, USA). The miRNA levels were quantified with a miScript SYBR Green Kit containing 10 × miScript Universal Primer (Qiagen, Germantown, USA). The RT-qPCR was performed by mixing 1 μl of template cDNA with 5 μl of 2 × QuantiTect SYBR Green PCR Master Mix (Qiagen, Germantown, USA), 10 × miScript Primer assay, and 10 × miScript Universal Primer in 10 μl of final volume. The PCR program consisted of an initial denaturation step at 95°C for 15 min, followed by 40 cycles of denaturation at 94°C for 15 s, a combined primer annealing-extension step at specific primer annealing temperatures for 30 s and then at 70°C for 30 s. A melting curve was produced afterwards by heating samples from 70°C to 95°C in 0.5°C increments for 5 s to confirm a single specific peak for each pair of primers. *U6* (Mondou et al., 2012; Abd El Naby et al., 2013) and *SNORD61* (Qiagen, Germantown, USA), previously shown to be stable in bovine embryos, were quantified to normalized mRNA expression levels using geNorm (Vandesompele et al., 2002).

### Cell Culture and Transfection

HEK293Ts or MDBKs were thawed and resuspended in Dulbecco Modified Eagle Media (DMEM) (Thermo Fisher Scientific, Waltham, USA) containing penicillin/streptomycin (100 U/ml) and 10% FBS (VWR, Radnor, USA). Culture occurred at 37°C, 5% CO_2_ in an incubator. MiR-10b mimics or control mimics were delivered into MDBKs using Hiperfect reagent (Qiagen, Germantown, USA) in Opti-MEM media (with a final concentration of 50 nM). SiRNA or siNTC was transfected into MDBKs using Lipofectamine 2000 (Invitrogen, Carlsbad, USA) according to the manufacturer’s instructions (with a final concentration of 500 nM). Twenty-four or 48 h after transfection, total RNA or protein was extracted for RT-qPCR or Western blotting (WB).

### Validation of *HOXA1* as a Target of miR-10b

To understand the mechanisms by which miR-10b induces apoptosis of embryonic cells, we used three computational algorithms, Targetscan, PicTar, and Miranda, to identify putative miR-10b targets in cattle. If a target was identified by all three algorithms, it was considered likely to be a miRNA target. Of the putative target genes identified in this way, *HOXA1* was chosen for further analysis because it was previously shown to be implicated in cell proliferation in human epithelial cells (Bitu et al., 2012) and cell growth, invasion, and migration in esophageal cancer cells (Li et al., 2017), which makes it of particular interest. The wild-type 3′UTR of *HOXA1* (594 bp) (NC_037331.1) containing the predicted miR-10b binding site was amplified and ligated into the psiCHECK2 vector (Promega, Madison, USA) *via* NotI and XhoI sites and subsequently confirmed by sequencing. To test whether the predicted miR-10b target site in the *HOXA1* 3′UTR is critical for the miR-10b-mediated repression of *HOXA1* expression, the seed sequence of the predicted miR-10b’s binding site was mutated. The primer sequences used for vector construction are listed in **Supplementary Table S1**.

HEK293T cells (70–80% confluency) were co-transfected with 500 ng of plasmid harboring wild-type or mutant sequences of the 3′UTR of *HOXA1* and 5 nM miR-10b mimics/control mimics using Lipofectamine 2000 in Opti-MEM media. Transfected cells were collected 24 h post-transfection and assayed using the Dual Luciferase reporter Kit (Promega, Madison, USA).

### Protein Isolation and Western Blot

Western blot was carried out using standard methods. Briefly, total protein was extracted from cultured cells 48 h after transfection using radioimmunoprecipitation lysis buffer consisting of 50 mM Tris–HCl (pH 7.5), 1% NP-40, 0.1% SDS, 0.5% sodium deoxycholate, 150 mM NaCl, and protease inhibitors. Before being loaded onto 10% SDS-polyacrylamide gels, the samples were denatured for 10 min at 100°C. Separated proteins were then transferred onto nitrocellulose membranes and subsequently blocked overnight with 5% non-fat milk in PBS with 0.1% Tween-20. Afterwards, membranes were incubated overnight with 1/1,000 rabbit anti-HOXA1 (Novus Biologicals, Abingdon, UK) or 1/1,000 rabbit anti-β-actin. After three washes, the membranes were incubated with HRP-conjugated goat anti-rabbit IgG (H+L) at room temperature for 2 h. Signals were detected by autograph using SuperSignal West Femto Maximum Sensitivity Substrate (Thermo Fisher Scientific, Waltham, USA).

### Cell Cycle Assays: PI Staining and Flow Cytometry

Forty-eight hours after transfection, MDBKs were collected by centrifugation, followed by fixation in ice-cold 70% ethanol at 4°C overnight. Then, the cells were stained with a final concentration of 50 µg/ml propidium iodide PI and 100 µg/ml RNase A in PBS. After 30 min in the dark, the stained cells were analyzed using Accuri^TM^ C6 flow cytometry (BD, Erembodegem, Belgium).

### Cell Viability Assays: WST-1 Colorimetric Assay

Cell viability was determined using the WST-1(4-(3-(4-iodophenyl)-2-(4-nitrophenyl)-2H-5-tetrazolio)-1,3-benzene disulfonate) (Merck, Kenilworth, USA). The assay was performed with ∼20,000 cells using 96-well plates. Forty-eight hours after transfection, 10 µl of WST-1 was added to 90-µl samples. The samples were then measured at 450 nm wavelength (570 nm as a reference wavelength) on an EZ read 400 microplate reader (Biochrom, Holliston, USA). After background subtraction, the viability was determined by comparing the absorbance values of samples.

## Statistical Analysis

The statistical analyses were performed using Student’s *t* test or ANOVA followed by Tukey’s test using GraphPad prism version 5. For each analysis, *P* < 0.05 was considered significant. The data are presented as mean ± S.D. and derived from at least three independent experiments.

## Results

### MiR-10b Mimics Can Be Taken Up by Bovine Embryos and Increase Apoptosis of Embryonic Cells

**Figure 1:**
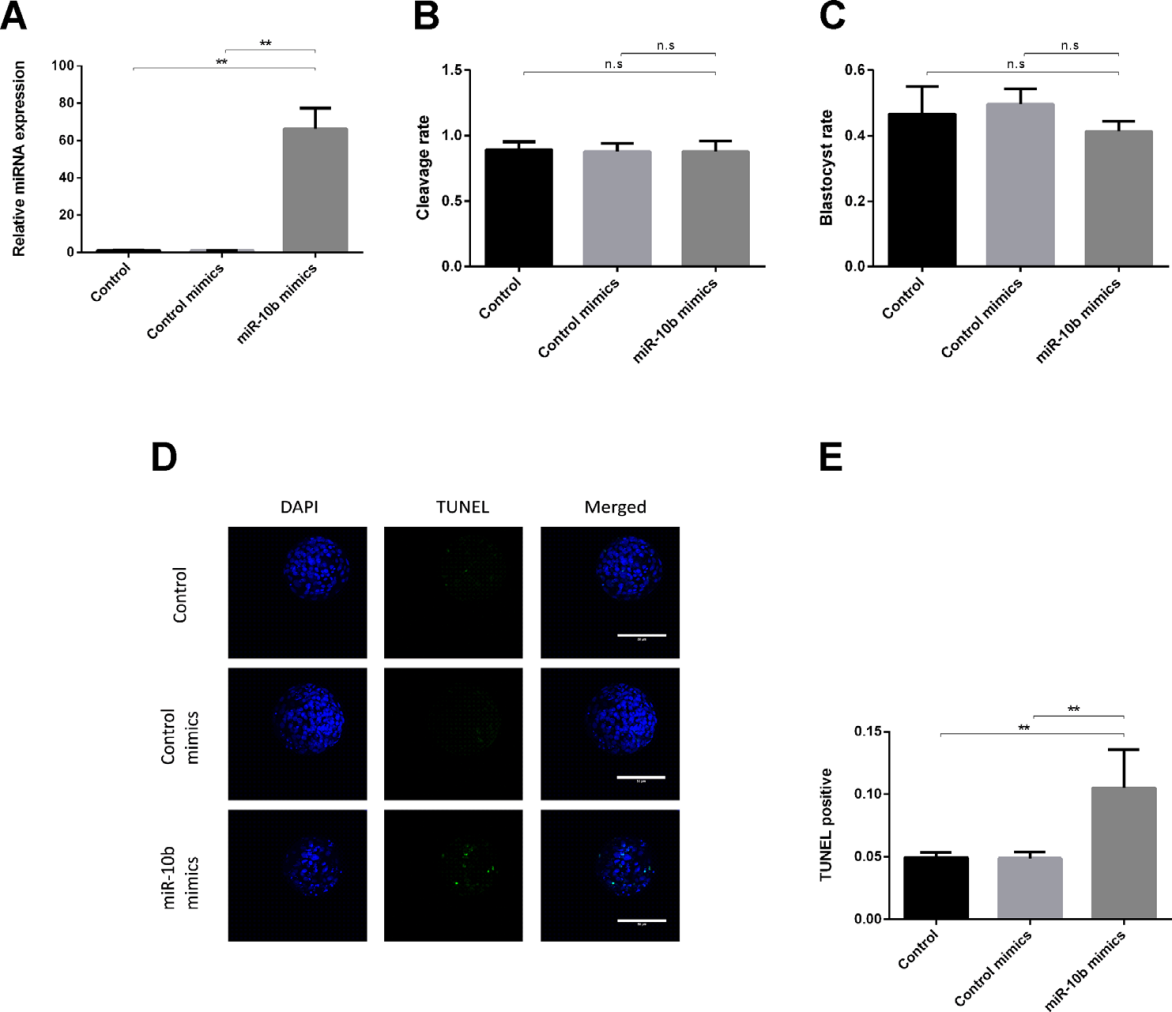
Effects of miR-10b mimics on embryo growth and apoptosis. **(A)** Embryos were treated with miR-10b mimics and the relative expression level of miR-10b was detected using RT-qPCR at 8 dpi. **(B** and **C)** Cleavage and blastocyst rate of embryos treated with miRNA mimics or control mimics were assessed. **(D** and **E)** Apoptosis rate of embryos was determined by TUNEL staining. The statistical analyses were performed using one-way ANOVA and data are presented as mean ± SD of three experiments (**P < 0.01; ns, no significance).

RT-qPCR results showed that miR-10b is indeed taken up by the embryos as its levels were noticeably higher (approximately 70 times) in the miR-10b mimics supplemented group compared with the control mimics group (**Figure 1A**). No significant difference was found in cleavage or blastocyst rate between the miR-10b mimics group and the control mimics group (**Figures 1B, C**). However, TUNEL staining showed a higher apoptosis rate in the miR-10b mimics group (10.52%) than in the control mimics group (4.88%) (**Figures 1D, E**).

### MiR-10b Regulates the Expression of HOXA1 Protein

**Figure 2:**
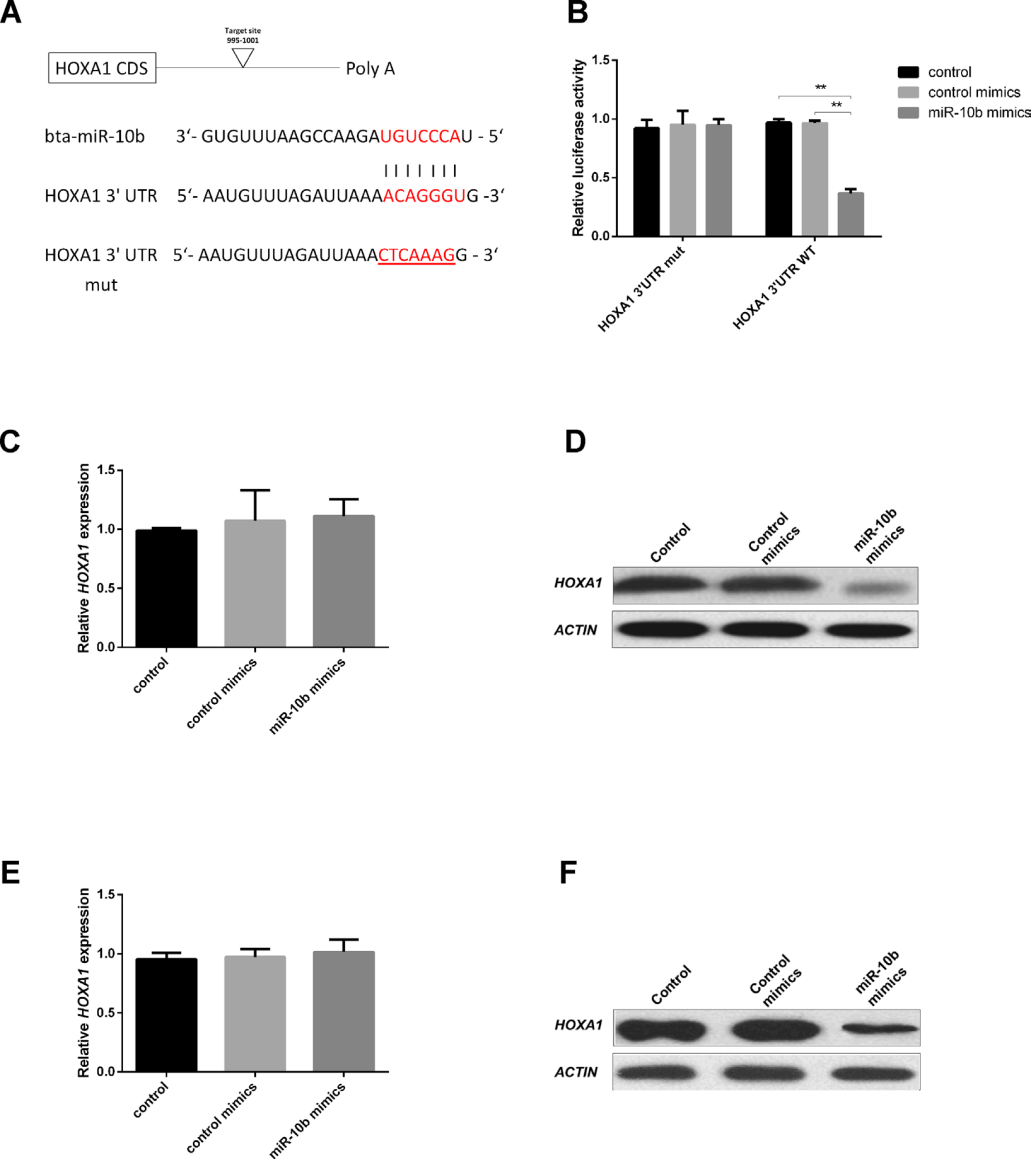
*HOXA1* is a direct target of miR-10b. **(A)** 3′UTR analysis of *HOXA1* containing putative regions that match the seed sequence of miR-10b, and the mutated nucleotides are underlined. **(B)** Overexpression of miR-10b inhibited Renilla luciferase activities. HEK-293T cells were cotransfected with 500 ng of reporter plasmid containing the MUT or WT-type UTRs and 5 nM miR-10b mimics. After 24 h, Renilla luciferase was normalized against firefly luciferase and then presented. **(C** and **D)** Embryos were treated with miR-10b mimics or control mimics and the relative levels of *HOXA1* were detected with RT-qPCR and WB. **(E** and **F)** MiR-10b mimics were transfected into MDBKs. After 48 h or 24 h, cells were harvested for RT-qPCR or WB. The statistical analyses were performed using ANOVA and data are presented as mean ± SD of three experiments (**P < 0.01).

The *HOXA1*-encoded mRNA contains a 3′UTR element that is complementary to the miR-10b seed sequence (**Figure 2A**). To evaluate whether miR-10b directly targets *HOXA1*, we constructed luciferase reporter plasmids with wild-type (psiCHECK2-*HOXA1*-WT-3′UTR) and a mutated 3′UTR (psiCHECK2-*HOXA1*-MUT-3′UTR). After co-transfecting the reporters with miR-10b mimics into HEK293T cells, we observed a clear reduction in the activity of the luciferase reporter gene fused to the wild-type *HOXA1* 3′UTR (63.5% reduction, **Figure 2B**).

After miR-10b mimics supplementation into embryo culture medium, no significant mRNA difference could be observed (**Figure 2C**), but a reduction of the endogenous HOXA1 protein was clear (**Figure 2D**). This indicates that miR-10b directly targets *HOXA1* and inhibits translation of the mRNA.

Since further functional analysis of miR-10b and *HOXA1* was out of practical necessity, performed using a bovine cell line (MDBK), the direct relationship between miR-10b and *HOXA1* was also analyzed on MDBKs. MiR-10b mimics were delivered into MDBKs with Lipofectamine 2000 and the expression of *HOXA1* was examined using RT-qPCR and WB. No significant differences were found at the mRNA level (**Figure 2E**), while HOXA1 protein levels were reduced in the miR-10b mimics group compared with the control mimics group (**Figure 2F**), in agreement with the results obtained on embryos. Other potential targets of miR-10b are listed in **Supplementary Table S2**. Among these potential target genes, *HOXD10* has been already proven to be a direct target of miR-10b and regulates cell proliferation in human glioblastoma cells and hepatocellular carcinoma cells (Su et al., 2001; Chisaka and Kameda, 2005). *HOXA3*, another member of the HOX family, was reported to regulate cell proliferation in mouse thymic epithelial cells and neural crest cells (Su et al., 2001; Chisaka and Kameda, 2005) and differentiation in human hematopoietic progenitor cells (Mahdipour et al., 2011).

### MiR-10b Mimics Result in Aberrant *DNMTs* Expression in Bovine Embryos

**Figure 3:**
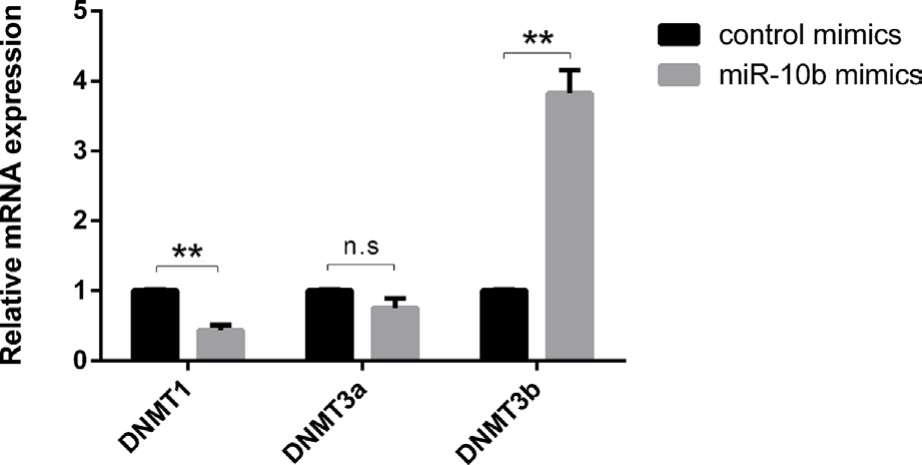
Effects of miR-10b mimics on *DNMTs* expression. Embryos were treated with miR-10b mimics and the relative expression levels of *DNMTs* were detected using RT-qPCR at 8 dpi. The statistical analyses were performed using two-way ANOVA, and data are presented as mean ± SD of three experiments (**P < 0.01; ns, no significance).

Recent studies have shown that epigenetic changes such as DNA methylation and miRNAs play crucial roles in embryonic development (Greenberg et al., 2016; Liu et al., 2016; Okada and Yamaguchi, 2017). However, the interaction mechanisms between miRNAs and DNA methylation have remained largely unexplored. In this study, we examined expression of *DNMTs* after supplementation of miR-10b mimics into embryo culture media. The expression of *DNMT1* was significantly lower in the miR-10b mimics-treated group compared with the control mimics group, while *DNMT3b* expression was significantly higher in the miR-10b mimics group compared with the control mimics group. No significant differences were found in *DNMT3a* for embryos cultured with miR-10b mimics versus control mimics (**Figure 3**).

### Microinjection of si*HOXA1* Increases Apoptosis of Embryonic Cells and Downregulates *DNMTs* in Bovine Embryos

**Figure 4:**
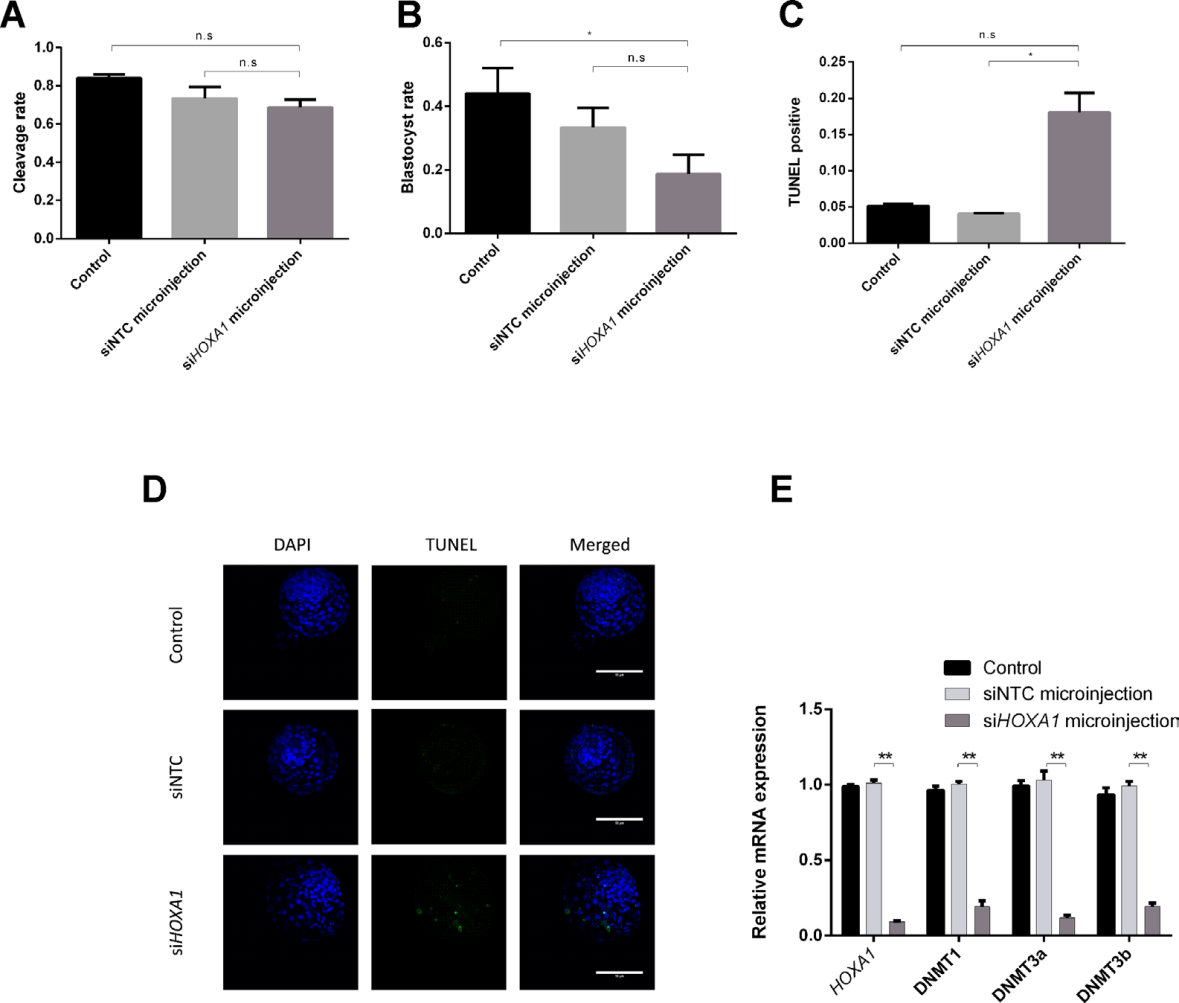
Effects of *HOXA1* in embryo growth, apoptosis, and *DNMTs*. Embryos were injected with si*HOXA1* and the cleavage rate **(A)** and blastocyst rate were assessed **(B)**; cell apoptosis was determined by TUNEL staining **(C** and **D)**; *HOXA1* and *DNMTs* expressions were evaluated using RT-qPCR **(E)**. The statistical analyses were performed using ANOVA and data are presented as mean ± SD of three experiments (*P < 0.05, **P < 0.01; ns, no significance).

To investigate the possible mechanisms of *HOXA1*’s function on embryos, blastocyst formation was determined and the expression of *DNMTs* in embryos was validated after si*HOXA1* microinjection. As presented in **Figure 4A**, the cleavage rate showed no significant difference between the si*HOXA1*-injected group and the siNTC-injected group, while the blastocyst rate was decreased in the si*HOXA1*-injected group compared with the siNTC-injected group, but the change did not reach statistical significance (**Figure 4B**). TUNEL staining showed that the apoptosis rate in the si*HOXA1*-injected group was 18.06%, while it was 4.07% in the siNTC-injected group (**Figures 4C, D**), meaning that the injection of si*HOXA1* increased apoptosis of embryonic cells.

We also examined expression of *HOXA1* mRNA and *DNMTs* mRNA after injecting si*HOXA1* into embryos. The expression of *HOXA1* was 10.8 times lower in the si*HOXA1*-injected group than in the siNTC-injected group (**Figure 4E**). The expression of *DNMT1* (5.2 times), *DNMT3a* (8.7 times), and *DNMT3b* (5.2 times) was found to be significantly decreased in the si*HOXA1* injected group compared to the siNTC group (**Figure 4E**).

### MiR-10b Overexpression and *HOXA1* Knockdown in MDBKs Decrease Cell Viability

**Figure 5:**
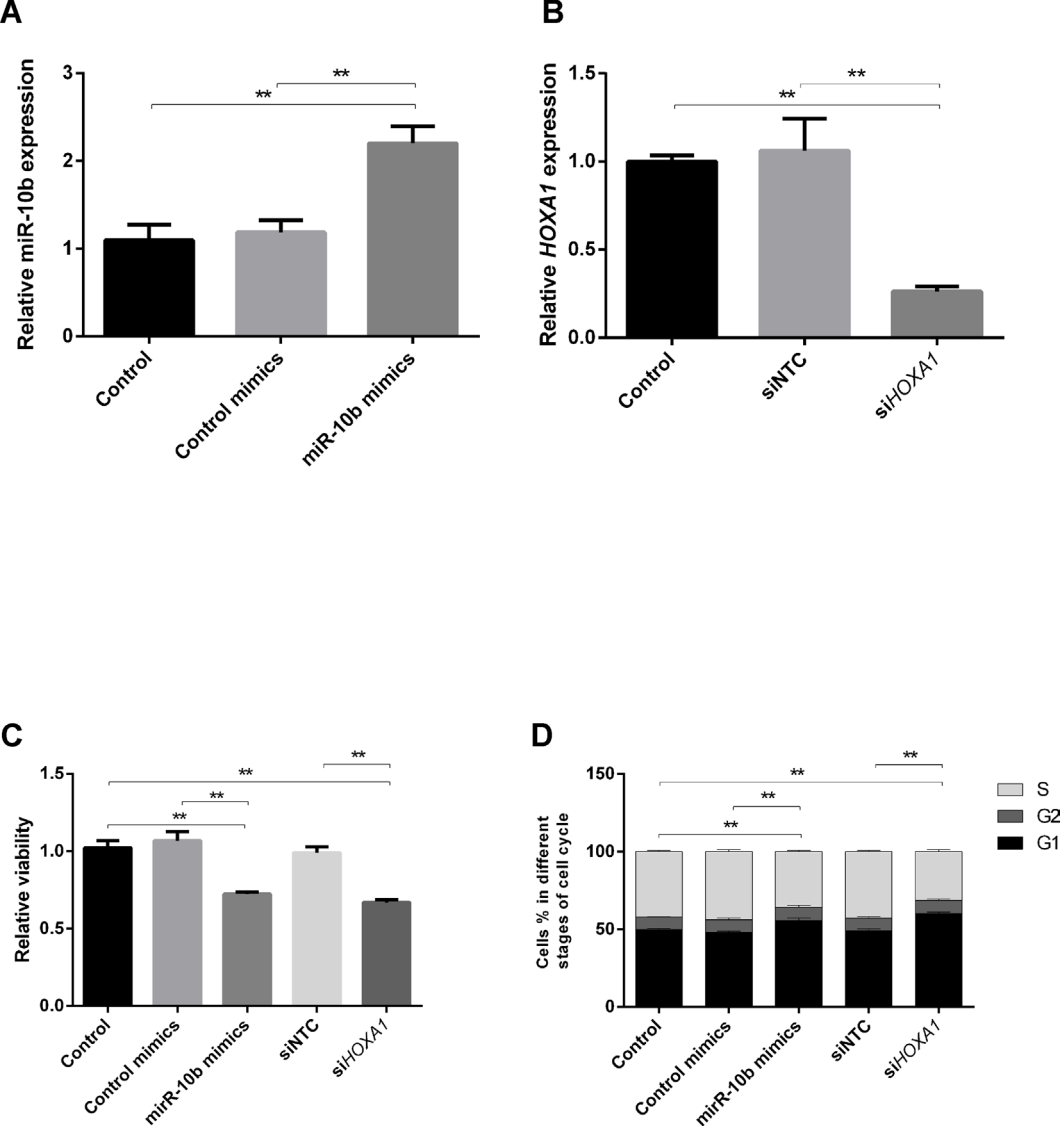
Effects of miR-10b overexpression and *HOXA1* knockdown on cell progression. **(A** and **B)** MDBKs were reverse transfected with miR-10b mimics or si*HOXA1* for 48 h. RT-qPCR was then performed to assess the expression of miR-10b and *HOXA1*. **(C** and **D)** MDBKs were reverse transfected with miR-10b mimics or si*HOXA1* for 48 h. **(C)** Cell viability was measured using the WST-1 assay, and **(D)** cell cycle assay by PI staining. Data are presented as mean ± SD of three experiments (**P < 0.01).

Although miR-10b has been shown to regulate cell progression in human and mouse, the regulatory relationship is still unclear in bovine. To further verify the above results, we explored the effect of miR-10b overexpression and *HOXA1* knockdown on cell progression using the bovine cell line MDBK. MDBKs were transfected with miR-10b mimics or si*HOXA1*, and their expression was assessed using RT-qPCR. As presented in **Figures 5A, B**, the expression of miR-10b was successfully increased in MDBKs by delivery of miR-10b mimics and *HOXA1* expression was significantly knocked down in MDBKs by transfection with si*HOXA1*.

The cellular metabolic activity, which indicates cell viability, was monitored using the WST-1 assay. As shown in **Figure 5C**, cell viability was reduced in miR-10b mimics-delivered cells (28%) compared with control mimics-delivered cells. Similarly, the inhibition of *HOXA1* significantly decreased the cell viability (34%).

### MiR-10b Overexpression and *HOXA1* Knockdown in MDBKs Slow Down the Cell Cycle

To elucidate the mechanism of growth inhibition by miR-10b overexpression and *HOXA1* downregulation, flow cytometry was used to analyze the cell cycle in MDBKs. The intensity of PI staining has a positive correlation with the number of cells. A higher proportion of cells in G1 indicates the slowing down of the cell cycle, while a higher proportion of cells in G2 and S stages indicates the promoting of the cell cycle. As shown in **Figure 5D**, cell cycle phase displayed a 7.66% increase of treated cells in the G1 phase and an 8.13% decrease in the S phase after the delivery of miR-10b mimics, indicating cell growth suppression. Similarly, knockdown of *HOXA1* resulted in a 10.86% increase in cell number in the G1 phase and an 11.48% decrease in the S phase compared with the siNTC-transfected group.

## Discussion

In our previous study (Lin et al., 2019), we have reported that several miRNAs were differentially released into conditioned media from bovine embryos with different developmental competence. One of those, miR-10b, was previously shown to be expressed in bovine embryos (Goossens et al., 2013), oocytes (Abd El Naby et al., 2013), follicles, and ovarian tissues (Huang et al., 2011; Gebremedhn et al., 2015). Several studies have shown that miR-10b plays important roles in cell apoptosis, cell proliferation, cell migration, and invasion in human cancer cells (Wang et al., 2007; Liao et al., 2014; Chen et al., 2016; Zhen et al., 2016; Zhu et al., 2016; Guan et al., 2018), mouse cells (Tan et al., 2018), and goat granulosa cells (Peng et al., 2016).

To further investigate how miR-10b negatively impacts bovine preimplantation bovine quality, we tested two possible mechanisms based on target gene prediction and literature.

Firstly, we tested *HOXA1* and verified it as a direct target of miR-10b. As one of the HOX family members, *HOXA1* is involved in various biological processes, including cell apoptosis and growth (Zhang et al., 2018). For instance, it was demonstrated that *HOXA1* can inhibit the migration, invasion and growth of HepG2 cells (Zha et al., 2012). Besides, forced expression of *HOXA1* in human mammary carcinoma cells resulted in increased proliferation and decreased apoptotic cell death in a Bcl-2-dependent manner (Zhang et al., 2003). In addition, *HOXA1* was found to enhance cell invasion, proliferation, and metastasis of prostate cancer cells (Wang et al., 2015). By microinjecting si*HOXA1* into zygotes, we found increased apoptosis in bovine embryos, an effect similar to after adding miR-10b mimics. The combination of these results with the observation that the HOXA1 protein level was decreased after supplementing miR-10b mimics into culture medium of embryos gives an indication that miR-10b might induce apoptosis of embryonic cells *via* targeting *HOXA1*. This could be in a Bcl-2-dependent manner as mentioned above, or through regulating other proteins involved in the apoptotic process, such as Bax, Bak, Bcl-xL, Fas, and FADD (Zhang et al., 2003).

Secondly, we tested if miR-10b exerts its negative effect on embryo development by interacting with the DNA methylation status. Since *DNMTs* are known to be involved in the maintenance of methylation patterns of genes (*DNMT1*) and de novo methylation (*DNMT3a* and *DNMT3b*), we investigated the mRNA levels of all three members after miR-10b mimics supplementation in culture medium. This resulted in a decrease in *DNMT1* expression, an increase of *DNMT3b* expression, and no effect on *DNMT3a*. The maintenance of methylation and de novo methylation are two distinct processes that are required for the establishment and mitotic inheritance of tissue-specific methylation patterns. *DNMT1* is recognized as the maintenance *DNMT* that copies methylation patterns after DNA replication as it has a preference for hemimethylated, rather than unmethylated DNA (Talbot et al., 1997). Loss of *Dnmt1* in mice has been reported to cause global DNA methylation loss and embryonic death (Tsumura et al., 2006). Moreover, loss of *DNMT1* in human colon cancer cell lines contributes to growth impairment (Rhee et al., 2002). *DNMT3b* is essential for early embryonic development and responsible for de novo methylation (Watanabe et al., 2002; Uysal et al., 2017). In fact, overexpression of *DNMT3b* was shown to result in aberrant DNA methylation in T-cell acute lymphoblastic leukemia (Poole et al., 2017) and to be significantly correlated with unfavorable prognosis in various human malignancies (Kim et al., 2006; Park et al., 2006; Vallböhmer et al., 2006; Wang et al., 2006; Lin et al., 2007; Xing et al., 2008).

Our data indicate an interaction between miRNA expression and DNA methylation, which is in agreement with other studies (Kim et al., 2014; Shivakumar et al., 2017; Wang et al., 2017). Taken together, the *DNMT1* downregulation and *DNMT3b* overexpression after overexpressing miR-10b found in the present study points to a link between aberrant DNA methylation and hampered development in bovine embryos. To our knowledge, this is the first study focusing on miRNAs regulation of DNA methylation in bovine embryos.

Since our results outlined above showed that miR-10b regulates both *HOXA1* and *DNMTs*, we further investigated the possible relationship between *HOXA1* and DNA methylation. Microinjection of si*HOXA1* clearly downregulated all three *DNMTs*, indicating that miR-10b may exert its inhibitory effect on *DNMT1* by regulating *HOXA1*, as *DNMT1* mRNA has no binding site for miR-10b (according to three computational methods: Targetscan, PicTar and Miranda) and hence is not a direct target of miR-10b. In a similar way, *DNMT3b* mRNA also has no binding site for miR-10b, which indicates that the upregulation of *DNMT3b* by miR-10b could be an indirect effect mediated by one or multiple other targets of miR-10b. Moreover, miR-10b overexpression and HOXA1 knockdown resulted in aberrant DNMT expression and an increased embryo apoptosis ratio. Previous findings in human cancer cells have shown that cell apoptosis and cell proliferation are related to DNA methylation, and DNA methylation can help inactivate apoptotic pathways at several points (Cho et al., 2011; Ye et al., 2016; Costa et al., 2017; Loginov et al., 2017).

Considering the fact that the compaction of embryos makes it difficult to use them for flow cytometry analysis, a complementary study regarding the effect of miR-10b overexpression and *HOXA1* knockdown on cell cycle was performed using the bovine cell line MDBK. The delivery of miR-10b mimics to MDBKs resulted in reduced cell viability and a high proportion at G1 stage. Similarly, the transfection of si*HOXA1* also led to reduced cell viability and a high proportion at G1 stage, indicating that miR-10b suppresses cell growth by targeting *HOXA1* and thus complementing the results obtained in the embryos.

Given the above results, bovine embryo-secreted miR-10b can be regarded as a potential biomarker for suppressed preimplantation developmental competence. miRNAs are gaining interest as potential biomarkers for diseases (Mitchell et al., 2008; Qu et al., 2017; Ma, 2018), embryo development in cattle (Kropp and Khatib, 2015), and embryo viability in human (Rosenbluth et al., 2014). According to the majority of studies, miRNAs are stable biomarkers. For instance, it was shown that miRNA levels remain remarkably stable when plasma is freeze–thawed multiple times or subjected to prolonged room temperature incubation (Mitchell et al., 2008). Besides, it was reported that miRNAs were stable frozen or refrigerated for 72 h and at room temperature for 24 h (McDonald et al., 2011). Apart from the stability, a biomarker should be easily detected. We have found miR-10b to be significantly higher expressed in the conditioned media of degenerate embryos compared to blastocysts in a previous study (Lin et al., 2019).

In this study, we examined the effects of embryo-secreted miR-10b on apoptosis and DNA methylation in bovine embryos. We conclude that miR-10b enhances apoptosis of embryonic cells *via* targeting *HOXA1*. Additionally, we found aberrant *DNMTs* expression after miR-10b mimics supplementation into embryo culture medium. As *DNMTs* are not direct targets of miR-10b, it probably exerts its effect on these genes through a network of other genes, among which is *HOXA1*.

## Data Availability

All data generated or analyzed during this study are included in this published article and its Supplementary Information file.

## Ethics Statement

All animal handlings were approved by the Ethical Committee of the Faculty of Veterinary Medicine (EC2013/118) of Ghent University. All methods were performed in accordance with the relevant guidelines and regulations.

## Author Contributions

XL performed the experiment and wrote the manuscript. KP helped to produce and stain embryos. KS contributed to the microinjection experiments. DD, BH, AS, and LP participated in the study design. All authors reviewed the manuscript.

## Conflict of Interest Statement

The authors declare that the research was conducted in the absence of any commercial or financial relationships that could be construed as a potential conflict of interest.
