# Supplementary material for: Bta-miR-10b Secreted by Bovine Embryos Negatively Impacts Preimplantation Embryo Quality
Source: Front Genet. 2019 Aug 22;10:757. doi: 10.3389/fgene.2019.00757 (PMC6713719; doi:10.3389/fgene.2019.00757)
Supplement: Supplementary file 1 [file Table_1.docx]

**Supplementary table S1. Sequences of primers for 3’UTR of *HOXA1* and mRNA quantitation.**

| Name | Sequence 5’-3’ |
| --- | --- |
| Btau*HOXA1* 3' UTR F' (restriction site in bold fonts) | TTGAC**CTCGAG**AAAGTCAAAAGAAACCCTCC |
| Btau*HOXA1* 3' UTR R' (restriction site in bold fonts) | ATTAA**GCGGCCGC**CTTAGTGGTGAAGTTGGTGC |
| Btau*HOXA1* 3' UTR mut-R1 (mutations underlined) | ATTTCTTTGAGTTTGGGAGGG |
| BtauHOXA1 3' UTR mut-F1 (mutations underlined) | CAAACTCAAAGAAATTGGAGAGTAT |
| Btau*HOXA1*-cds-F | ACAGCCCCTACGCGTTAAAT |
| Btau*HOXA1*-cds-R | CAATTTTCCCTGTTTTGGGAGGG |
| Btau*DNMT1*+F | CTACCAGTGCACCTTTGGCGT |
| Btau*DNMT1*-R | GTGCGAACACATGCAACGGCT |
| Btau*DNMT3a*+F | GCATTGTGTCTTGGTGGATG |
| Btau*DNMT3a*-R | CTTGTTGTAGGTGGCCTGGT |
| Btau*DNMT3b*+F | AAGACCGGCCTTTCTTCTGGATGT |
| Btau*DNMT3b*-R | TGTGAGCAGCAGACACTTTGATGG |
| Btau*GAPDH*-F | TTCAACGGCACAGTCAAGG |
| Btau*GAPDH*-R | ACATACTCAGCACCAGCATCAC |
| Btau*YWHAZ*-F | GCATCCCACAGACTATTTCC |
| Btau*YWHAZ*-R | GCAAAGACAATGACAGACCA |

**Supplementary table S2. Predicted target genes of miR-10b.**

| ANK3 | CAMK2G | GATA6 | NR5A2 |
| --- | --- | --- | --- |
| ANKFY1 | CECR6 | GTF2H1 | SDC1 |
| ARIH2 | CNNM4 | HAS3 | SLC38A2 |
| ARNT | CSMD1 | HOXA1 | SMAP1 |
| ARRDC3 | CTNNBIP1 | HOXA3 | SON |
| BACH2 | DAZAP1 | HOXD10 | TBX5 |
| BAZ2B | EBF2 | MAPRE1 | TFAP2C |
| BCL6 | ELOVL6 | NCOA6 | TMOD1 |
| BDNF | EPHA4 | NCOR2 | TRIM2 |
| BICD2 | ESRRG | NFAT5 | XRN1 |
|  |  |  | ZMYND11 |
